# Correlation of serum homocysteine and previous history of gestational diabetes mellitus

**DOI:** 10.1186/2251-6581-12-34

**Published:** 2013-07-02

**Authors:** Sudabeh Alatab, Hossein Fakhrzadeh, Farshad Sharifi, Mojde Mirarefin, Zohreh Badamchizadeh, Maryam Ghaderpanahi, Arash Hossein-nezhad, Bagher Larijani

**Affiliations:** 1 Elderly Health Research Center, Endocrinology and Metabolism population Sciences Institute, Tehran University of Medical Sciences, North Karegar Avenue, Dr Shariati Hospital, 5th floor, Tehran, 1411413137 Iran; 2 Endocrinology and Metabolism Research Center, Endocrinology and Metabolism Research Institute, Tehran University of Medical Sciences, Tehran, Iran

**Keywords:** Gestational diabetes mellitus, Homocysteine, Diabetes mellitus

## Abstract

**Background:**

Gestational diabetes mellitus (GDM) is a common pregnancy condition. In this study, the risk of having a history of previous GDM (pGDM) on serum homocysteine level was assessed

**Methods:**

Biomedical parameters, serum homocysteine, Insulin, homeostatic model assessment (HOMA) in women with (n = 52) and without pGDM (n = 51) were assessed. According to their current status of Oral Glucose Tolerance Test (GTT), the participants in each group were divided into two subgroups of normal or impaired GTT.

**Results:**

Mean serum homocysteine in normal women was 8.56 ± 3.19 vs 11.44 ± 7.34 μmol/L (p < 0.01) in women with pGDM. Two groups had significant differences in respect to serum insulin levels (8.35 ± 5.12 vs 12.48 ± 5.44, p < 0.002), and HOMA-IR (1.90 ± 1.30 vs 2.91 ± 1.30, p < 0.002). In women without pGDM, serum homocysteine in normal and impaired GTT were 7.60 ± 1.69 and 10.52 ± 3.65 μmol/L (p = 0.03), respectively, while in women with pGDM, the figures were 8.38 ± 2.52 and 14.00 ± 10.17 (p < 0.01), respectively. In multi regression analysis an association between history of GDM and homocysteine levels was presented (OR: 7.71, 95% CI: 1.67-35.42, p < 0.001).

**Conclusion:**

A trend of elevation of homocysteine is presented in women with pGDM, that is more prominent in women with impaired GTT, and shows a significant correlation with history of GDM. Further studies with larger sample size are suggested.

## Background

Gestational diabetes mellitus (GDM) is a common condition which is defined as a different degree of the glucose intolerance that begins or is first detected during pregnancy. GDM affects between 2% and 5% of pregnant women in the united state [1]. In Iran it complicates 4-8% of pregnancies [2]. The pathogenesis of GDM is very similar to that of type 2 diabetes (T2D), in which both pancreatic insulin release and chronic insulin resistance have roles. GDM and impaired glucose tolerance during pregnancy are shown to be associated with future metabolic dysfunction and diabetes separate from other clinical risk factors [3]. The GDM patients may also show early markers of vascular disease such as endothelial dysfunction [4] which may make them susceptible to premature atherosclerosis and coronary heart disease.

Hyperhomocysteinemia, as a major independent risk factor for vascular disease, has been found in some studies to be associated with clinical conditions of insulin resistance [5–8]. In a study by Meigs et al. [7] hyperhomocysteinemia was associated with hyperinsulinemia. The authors claimed that hyperhomocysteinemia may partially account for an increased risk of cardiovascular disease seen in patients with insulin resistance. Though in some studies no or negative correlation was found between hyperhomocysteinemia and insulin resistance syndrome [9].

As we mentioned here the diagnosis of GDM signals a greater risk of developing diabetes. On the other hand, some studies points that hyperhomocysteinemia may be involved in development of insulin resistance conditions. Therefore in this study we aimed to investigate the association between having a history of previous GDM (pGDM) and hyperhomocysteinemia. We also sought to see the correlation between hyper hyperhomocysteinemia with insulin resistance condition. To perform this, we measured the serum levels of homocysteine in women with and without pGDM and evaluated to see that whether insulin resistance could exceed what that has been observed in normal pregnancy.

## Methods

### Study population

This study was performed in the Endocrinology and Metabolism Research Center of Tehran University of Medical Sciences. Participants were randomly drawn from the list of pregnant women who attended the obstetrics clinic at Dr. Shariati Hospital for regular prenatal care between 2005–2009. A total of 51 non-pregnant women with history of GDM (pGDM) and 52 non-pregnant unaffected women as control subjects with a mean of 4 years after their index pregnancy were enrolled. Two groups were matched based on their ages. The protocol of study was approved by the ethics committee of Tehran University of Medical Sciences and the participants signed their informed consent at the time of recruitment.

All of the participants underwent physical examination and anthropometric evaluations at the time of recruitment. By means of a special questionnaire, the maternal data including age, parity, habits and pregnancy events were collected. The inclusion criteria were defined as non menopause non pregnant women, aged between 20–44 years old. Patients who had pre-existing hypertension, symptomatic cardiovascular disease, using vitamin B supplements, current or previous smokers, or had a medical condition that influenced the homocysteine level (chronic renal failure, hypothyroidism, a history of breast or ovarian cancer) were excluded from the study.

Participants were stratified based on American Diabetes Association recommendation [10]. Those with fasting blood sugar (FBS) < 126 mg/dl and 2-h post glucose load < 140 mg/dl categorized as normal glucose tolerance (N-GTT) and patients with FBS < 126 mg/dl and 2-h post glucose load ≥ 140 mg/dl as impaired glucose tolerance (I- GTT).

Weight and height were measured. BMI was calculated by dividing weight (kg) by square of height (m). Blood pressure was measured with an automatic sphygmomanometer in the sitting position.

### Laboratory measurement

The blood sample for biochemical parameters were taken after an overnight fasting. Patients then underwent a 75- g oral glucose tolerance test at 2 h after glucose load, the blood samples were collected again. FBS, triglyceride (TG), total cholesterol, HDL cholesterol and LDL cholesterol were measured by enzymatic method (Pars Azmoon, Iran). Serum folate and vitamin B_12_ were measured by Radioimmunoassay (RIA assay, Simul TRAC, ICN Pharmaceutical). Homocysteine was measured by HPLC method. Plasma insulin was determined by immunoassay (ELISA) using a Bioscience kit (Monobind kit, Monobind Inc., Lake Forest, CA, USA). Homoeostasis model assessment (HOMA -IR) was calculated as [fasting plasma glucose (mmol/L) x fasting insulin (μU/mL) / 22.5].

### Statistical analysis

Statistical analysis was performed using Statistical Package for the Social Sciences (SPSS, version 18, Chicago, IL, USA). Values less than 0.05 were considered significant. Normality of values was tested by Kolmogorov- Smirnov test. Comparison of clinical and biochemical variables between groups were tested by t-test. Evaluation of homocysteine concentration according to glucose tolerance test status was done using one way ANOVA. Multiple logistic regression analysis was performed with categorized homocysteine (homocysteine > 14 vs ≤ 14) [11] as a dependent variable, and GDM status as an independent variable. Model was adjusted for serum folic acid, vitamin B12, FBS, TG, Cholesterol, LDL, insulin, diastolic blood pressure and HOMA-IR.

## Results

Table 1 shows the demographic and biochemical characteristics of participants. The mean ages of participants in control and pGDM groups were 32.23 ± 6.14 and 32.27 ±5.01 respectively. No significant difference in respect to FBS and B12 and folate levels was observed between two groups, while diastolic blood pressure, cholesterol, LDL, serum insulin, homocysteine, and HOMA-IR were significantly higher in women with pGDM (Table 1).

**Table 1:** **Anteropometric and biochemical characteristics of participants by GDM status**

GDM status	pGDM	Control	P
Variables	n = 51	n = 52	
Age (year)	32.27 ± 5.01	32.23 ± 6.14	0.97
BMI (kg/m^2^)	28.12 ± 3.57	27.58 ± 5.69	0.66
FBS (mg/dl)	100.47 ± 31.90	92.15 ± 14.75	0.21
TG (mg/dl)	127.90 ± 65.84	109.12 ± 45.50	0.19
Cholesterol (mg/dl)	195.67 ± 33.86	176.50 ± 37.84	0.02
LDL cholesterol (mg/dl)	108.27 ± 24.13	92.27 ± 24.71	< 0.001
HDL cholesterol (mg/dl)	50.00 ± 10.13	50.50 ± 10.24	0.83
Serum insulin	12.48 ± 5.44	8.35 ± 5.12	< 0.001
Serum creatinine (mg/dl)	0.80 ± 0.09	0.82 ± 0.11	0.34
Serum B12 (pg/ml)	437.67 ± 131.35	401.46 ± 154.93	0.31
Serum folic acid (ng/ml)	5.44 ± 2.69	5.12 ± 2.62	0.65
Serum Homocysteine (μmol/L)	11.44 ± 7.34	8.56 ± 3.19	0.01
Systolic Blood Pressure (mmHg)	119.47 ± 13.51	116.07 ± 14.19	0.30
Diastolic Blood Pressure (mmHg)	76.66 ± 10.90	71.69 ± 8.59	0.04
HbA1c (%)	5.42 ± 1.29	5.22 ± 0.67	0.49
Uric acid	4.87 ± 1.20	4.67 ± 1.08	0.48
HOMA-IR	2.91 ± 1.30	1.90 ± 1.30	<0.001

To evaluate the possible effects of pGDM on insulin resistance condition, each group of participants were divided based on whether currently they have glucose intolerance or not. Therefore in group of women with pGDM, subjects were divided into two groups of N-GTT andI-GTT. The women without pGDM were also divided into two subgroups of N-GTT and I-GTT (Table 2).

**Table 2:** **Serum homocysteine concentration according to glucose tolerance test status**

	pGDM		P	Control		P
	(n = 51)		value	(n = 52)		value
	N-GTT	I-GTT		N-GTT	I-GTT	
	(n = 39)	(n = 12)		(n = 45)	(n = 7)	
Homocysteine(μmol/L)	8.38 ± 2.52	14.00 ± 10.17	<0.01	7.60 ± 1.69	10.52 ± 3.65	0.03

Looking at the patients in this way, we observed a significant trend of increase in serum homocysteine concentration in both groups of women from N-GTT toward I-GTT (Table 2). In multiple logistic regression analysis, there was a significant association between homocysteine concentration and pGDM. Following adjustment for VitB12, folic acid, insulin, cholesterol, diastolic blood pressure, FBS, and TG, the significant relation was still present (Table 3).

**Table 3:** **Coefficients of homocysteine association with GDM status in a multiple logistic regression mode**

	Multivariate adjusted OR (95% CI)	P
pDGM	7.71 (1.67-35.42)	0.00
Vit B12	0.99 (0.99-1.00)	0.28
Folic acid	1.15 (8.23-1.62)	0.40
Insulin	0.89 ( 0.75-1.06)	0.21
Cholesterol	0.97 (0.94-0.99)	0.03
Diastolic BP	0.99 (0.92-1.08)	0.95
FBS	1.16 (1.08-1.25)	0.00
TG	1.00 (0.98-1.02)	0.54

Adjusted for: *VitB12*, folic acid, insulin, cholesterol, diastolic blood pressure, *FBS*, and *TG*.

## Discussion

Our first observation in this study was an early stage of insulin resistance in women with pGDM manifested by higher levels of fasting insulin, and HOMA IR compared to control group. The failure of pancreatic B cell function plays an important role in the pathogenesis of insulin resistance. Previous studies showed that impaired insulin secretion is the key in conversion from normal glucose tolerance to impaired glucose tolerance and finally diabetes [12, 13]. Although the proper measurement of B cell function, which is necessary for evaluation of insulin release,was out of scope of this study, but we couls state that a glucose impairment existed in our pGDM population. According to Bonora et al. [14], a HOMA -IR score ≤ 2.06 could be found in normal non-diabetic population. In the absence of local reference data for HOMA -IR score, we may assume a value of greater than 2.0 to represent insulin resistance condition. In our study, the HOMA -IR. of the pGDM was 2.91 compared to 1.90 in women without pGDM, which could be translated into that women with pGDM suffer from a state of insulin resistance. Moreover, we observed that women with pGDM had higher rate of I-GTT than non GDM subjects (23.5% vs 13.4). Similarly, and more recently, Molęda and his colleagues [15] reported a lower rate of normoglycaemia, at 60 and 2 h after glucose load, in women who had GDM history (57%) within the last 5–12 years compared to non-GDM women (88%).

Having a higher levels of homocyteine in GDM patients is reported by previous studies [16, 17]. Similarly, we found a significant difference in levels of serum homocysteine in a way that pGDM group had higher concentration of homocysteine. Meanwhile when the subjects in each group were divided based on their state of glucose metabolism, no significant changes was presented between pGDM and non GDM groups who had N-GTT, while this difference between I-GTT groups were remarkable. In humans, homocysteine is formed during the metabolism of methionine and is metabolized by re-methylation or trans-sulphuration, in which both vitamin B6 and B12 work as the cofactors. The postulation that vitamin status of participants might influence the result is unlikely, since the deficiencies and significan difference of folate and B_12_ were absent in our groups. Glomerular filtration rate as an indicator of the renal function is another independent determinant of homocysteine concentration [18]. Since the mean of GFR between two groups were similar (data not shown), it is unlikely that the higher levels of homocysteine is the result of more progressive kidney damage and more decreased clearance of homocysteine. Therefore, the weight of evidence from the present study may indicate that variations in plasma total homocysteine concentrations could be dependent to insulin sensitivity state, howeverwith this analysis, we can not make apparent the pathophysiological reason behind increased homocysteine in IGGT women.

In this study we found an association between history of GDM and high levels of homocysteine. We can not comment on that whether high levels of homocysteine are caused by the previous presence of impaired glucose tolerance in GDM subjects or patients who developed GDM had higher succeptibility to have a higher levels of homocysteine. Eitherway, presence of abnormalities of glucose metabolism are an indicator a manifestation of the clustering of several metabolic abnormalities, including dyslipidemia, hypertension, insulin resistance, and, therefore, increased cardiovascular risk. On the otherhand, epidemiological, retrospective as well as data from prospective studies support an association between elevated homocysteine levels and increased risk of cardiovascular disease [19]. It has been shown that high levels of plasma homocysteine increase the oxidative stress and decrease the NO dependent relaxation of endothelium [20]. Taking together, one can assume that having a history of GDM may present a multiple risk for cardiovascular disease since it could increase the risk of impairment in glucose metabolism and also development of higher levels of homocysteine.

We understand that our study has some limitation. low number of case as well as absence of detailed dietary information regarding the ingestion of a diet with a high insulinaemic index is among our limitations.

## Conclusion

In conclusion we performed a case–control study in women with and without history of GDM and observed a trend of increase in serum levels of homocysteine in pGDM. Further analysis showed that women with I-GTT had a higher levels of homocysteine. Whether this elevation was occurred during the course of development of glucose metabolism impairment and insulin resistance or is a causal factor needs to be evaluated. A significant correlation of homocysteine with history of GDM is an interesting observation of this study that calls for further studies with larger sample size. Moreover evaluation of endothelial function using flow mediated dilatation (FMD) in pGDM women with high levels of homocysteine would be interesting

## Abbreviations

GDM: Gestational diabetes mellitus
FBS: Fasting blood sugar
GTT: Glucose tolerance test
N-GTT: Normal glucose tolerance test
I-GTT: Impaired glucose tolerance test
TG: Triglyceride
HDL: High density lipoprotein
LDL: Low density lipoprotein
HOMA: Homoeostasis model assessment.
